# Supplementary material for: Hippocampus, Retrosplenial and Parahippocampal Cortices Encode Multicompartment 3D Space in a Hierarchical Manner
Source: Cereb Cortex. 2018 Mar 15;28(5):1898–909. doi: 10.1093/cercor/bhy054 (PMC5907342; doi:10.1093/cercor/bhy054)
Supplement: Supplementary Data [file bhy054suppl_1.zip › KimMaguireLegendSuppleFig4.docx]

**Supplementary Figure 4.** Exact location (or associated painting) encoding regions. (A) The whole brain contrast “same corner, same room < different corner or different room” was searched within the voxels that showed no difference between the “same corner, different room” (SCDR), “different corner, same room” (DCSR) and “different corner, different room” (DCDR). This revealed a cluster of activity in the posterior cingulate cortex (T(29)=6.47, p=0.011) and one active voxel in the vicinity of the putamen (T(29)=6.1, p=0.028). (B) Comparison of the mean activity for the four experimental conditions at the peak voxels. The “same corer, same room” (SCSR) condition showed the lowest activity and all three other conditions (SCDR, DCSR, DCDR) were similar, as would be expected from the definition of the contrast. Error bars are SEM adjusted for a within-subjects design (Morey, 2008).
