# Supplementary material for: Hippocampus, Retrosplenial and Parahippocampal Cortices Encode Multicompartment 3D Space in a Hierarchical Manner
Source: Cereb Cortex. 2018 Mar 15;28(5):1898–909. doi: 10.1093/cercor/bhy054 (PMC5907342; doi:10.1093/cercor/bhy054)
Supplement: Supplementary Data [file bhy054suppl_1.zip › KimMaguireLegendSuppleFig1.docx]

**Supplementary Figure 1.** Two views associated with each room. Participants could be transported to the room from two opposite ends of the corridor, (A) facing a floor sign wall or (B) facing a staircase. This created two visually distinctive approaches for each room.
