# Supplementary material for: Hippocampus, Retrosplenial and Parahippocampal Cortices Encode Multicompartment 3D Space in a Hierarchical Manner
Source: Cereb Cortex. 2018 Mar 15;28(5):1898–909. doi: 10.1093/cercor/bhy054 (PMC5907342; doi:10.1093/cercor/bhy054)
Supplement: Supplementary Data [file bhy054suppl_1.zip › KimMaguireLegendSuppleFig2.docx]

**Supplementary Figure 2.** Room and view encoding. Even when participants visited the same room from an approach with a different view, the fMRI signal was less than when visiting a different room (“same room, different view” < “different room”), suggesting the existence of abstract room information that is not explained by a particular view. However, RSC_R, PHC_R and postHC_R showed additional view dependency (“same room, same view” < “same room, different view”). Error bars are SEM adjusted for a within-subjects design (Morey 2008). *p<0.05.
