# Supplementary material for: Hippocampus, Retrosplenial and Parahippocampal Cortices Encode Multicompartment 3D Space in a Hierarchical Manner
Source: Cereb Cortex. 2018 Mar 15;28(5):1898–909. doi: 10.1093/cercor/bhy054 (PMC5907342; doi:10.1093/cercor/bhy054)
Supplement: Supplementary Data [file bhy054suppl_1.zip › KimMaguireLegendSuppleFig3.docx]

**Supplementary Figure 3.** Anatomical ROIs for the hippocampus (A) and retrosplenial cortex (B) used for the small-volume correction. These ROIs were manually delineated on the group-averaged structural MRI scan from a previous, independent, study of 3D space representation (Kim et al. 2017).
