# Supplementary figures and images for: Hippocampus, Retrosplenial and Parahippocampal Cortices Encode Multicompartment 3D Space in a Hierarchical Manner
Source: Cereb Cortex. 2018 Mar 15;28(5):1898–909. doi: 10.1093/cercor/bhy054 (PMC5907342; doi:10.1093/cercor/bhy054)

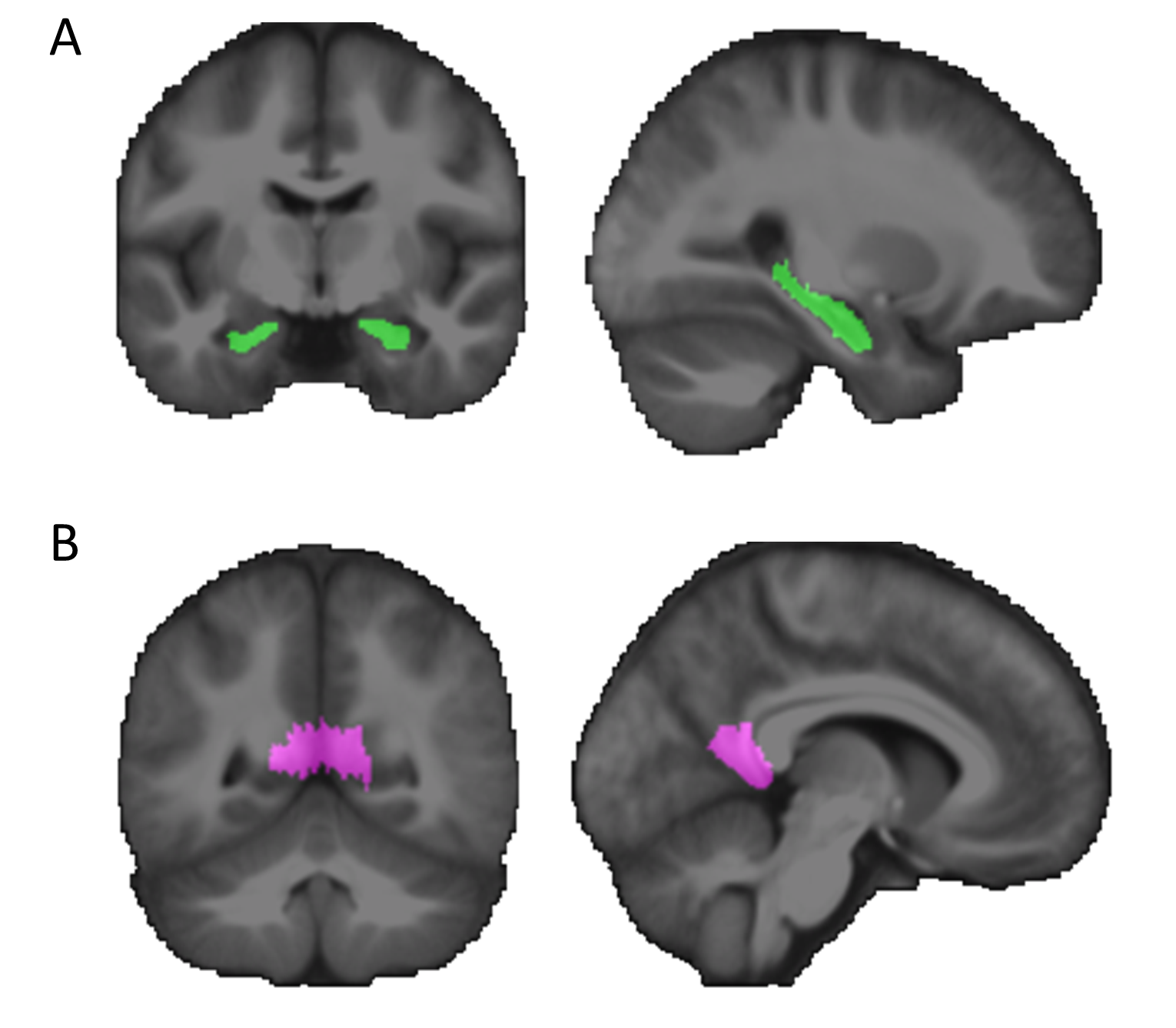

Supplement: Supplementary Data [file bhy054suppl_1.zip › KimMaguireSuppleFig3.tif]

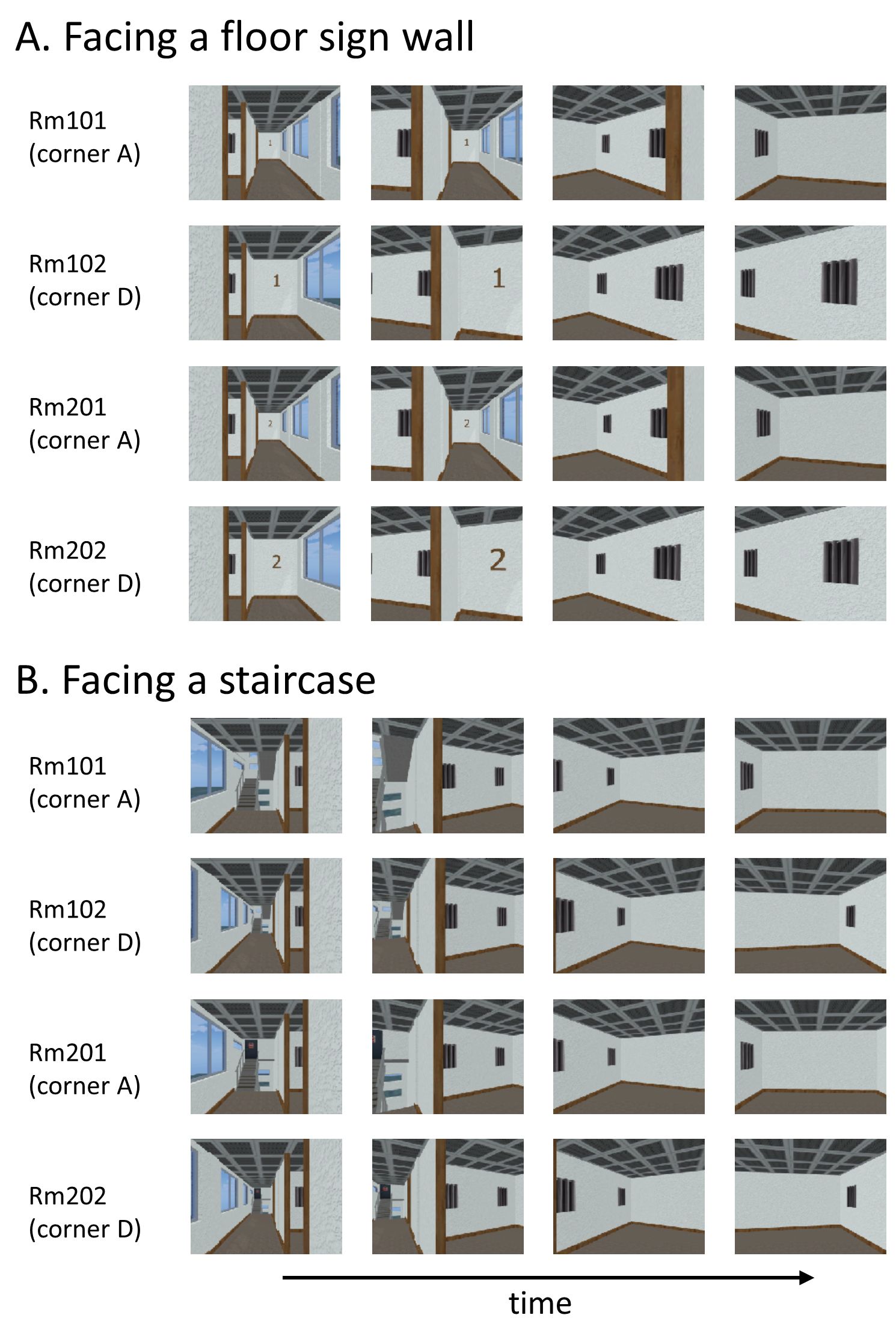

Supplement: Supplementary Data [file bhy054suppl_1.zip › KimMaguireSuppleFig1.jpg]

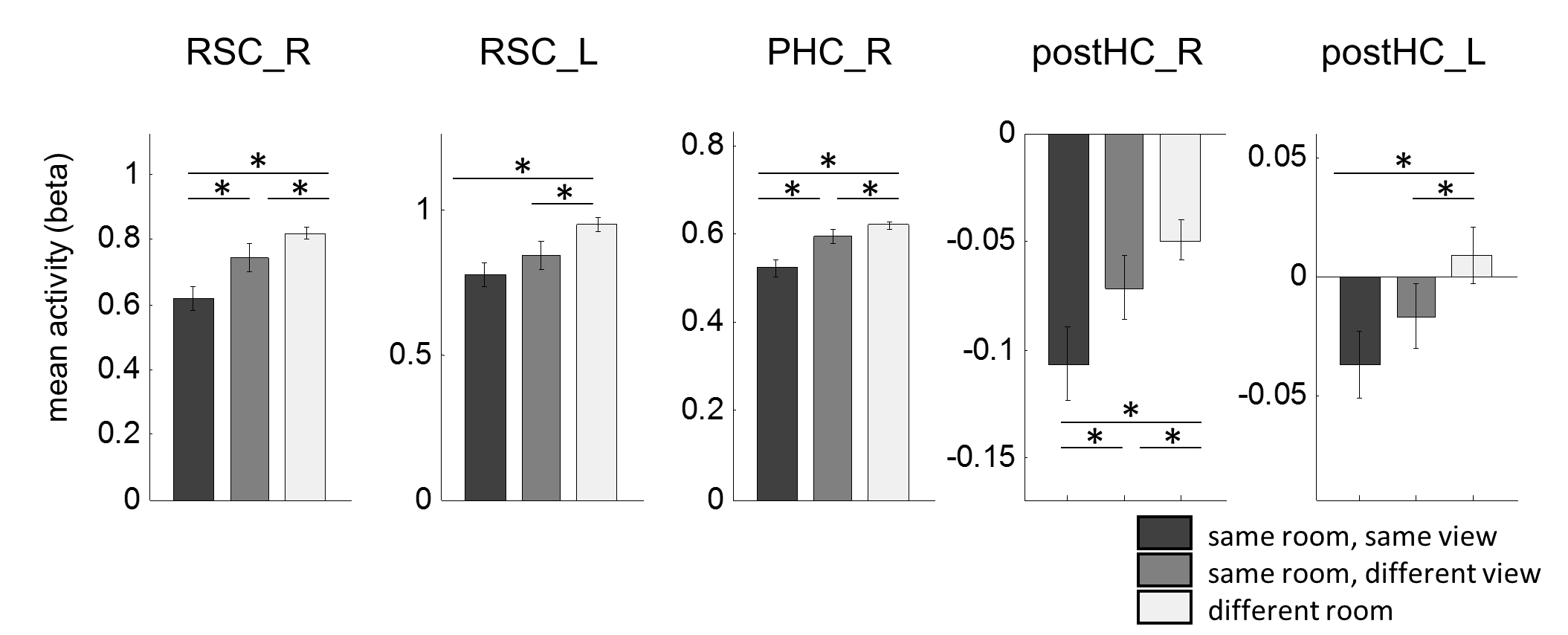

Supplement: Supplementary Data [file bhy054suppl_1.zip › KimMaguireSuppleFig2.tif]

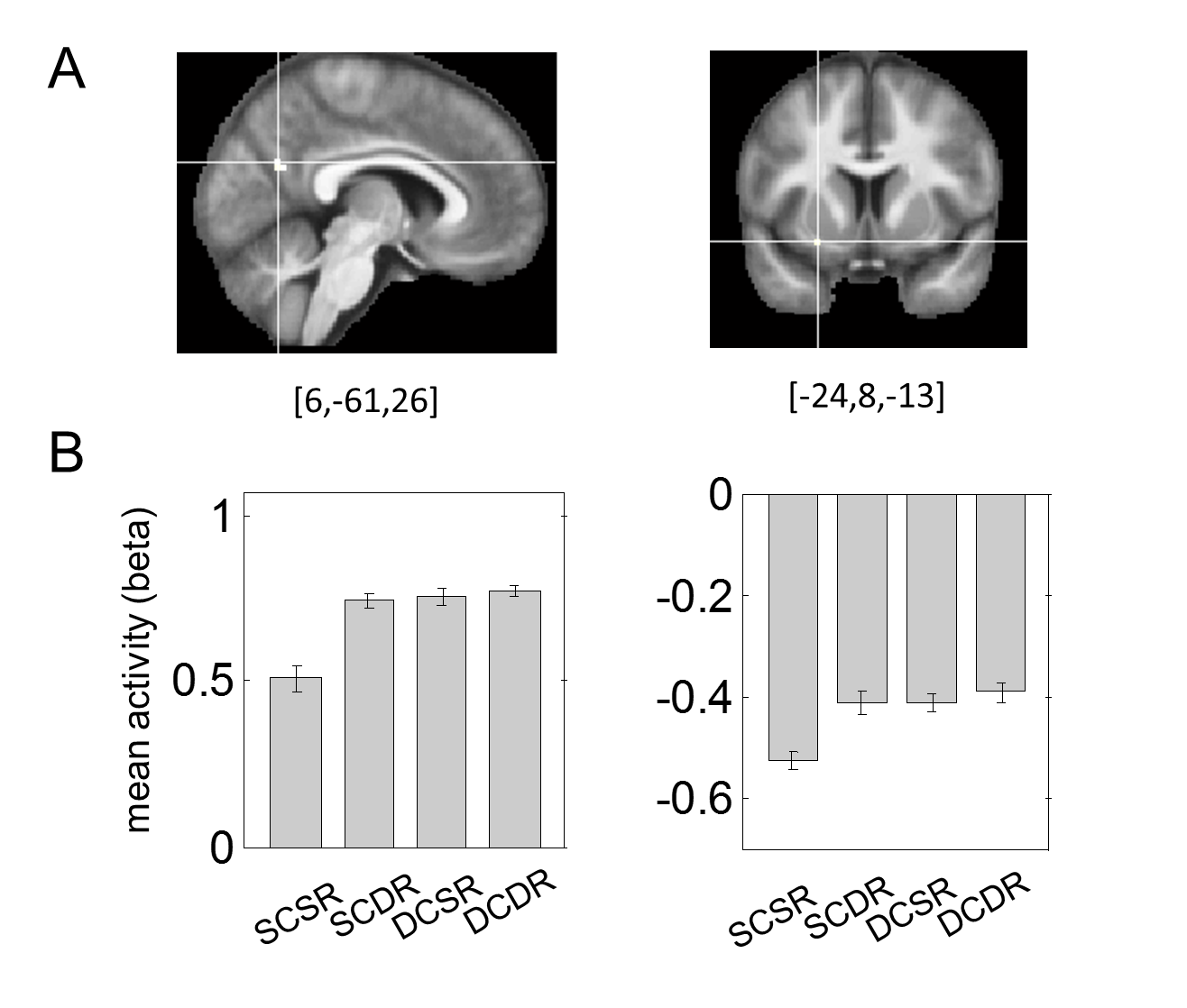

Supplement: Supplementary Data [file bhy054suppl_1.zip › KimMaguireSuppleFig4.tif]
